# Coenzyme Q10 supplementation therapy for 2 children with proteinuria renal disease and *ADCK4* mutation: Case reports and literature review

**DOI:** 10.1097/MD.0000000000008880

**Published:** 2017-11-27

**Authors:** Chunyue Feng, Qiong Wang, Jingjing Wang, Fei Liu, Huijun Shen, Haidong Fu, Jianhua Mao

**Affiliations:** a Department of Nephrology, The Children Hospital of Zhejiang University School of Medicine, Hangzhou; b Department of Nephrology, The Second Hospital of Jiaxing Municipal, Jiaxing, Zhejiang Province, China.

**Keywords:** *ADCK4* mutation, coenzyme Q10, mitochondrial nephropathy, proteinuria

## Abstract

**Rationale:**

Mitochondrial nephropathy has a poor prognosis and often progresses to the end-stage renal disease. Renal pathology often is focal segmental glomerulosclerosis (FSGS) and does not respond to steroid therapy or immunosuppressive therapy. Some patients are benefited from the therapy of coenzyme Q10, which affect the synthesis pathway of coenzyme Q10.

**Patient concerns:**

Herein, we report 2 cases of children with proteinuria renal disease with *ADCK4* mutation.

**Diagnoses:**

Proteinuria renal disease with *ADCK4* mutation.

**Interventions:**

Compound heterozygous mutation in *ADCK4* gene were detected with next-generation sequencing and confirmed by Sanger sequencing. Both of the patients were given coenzyme Q10 supplementation therapy.

**Outcomes:**

The first patient showed a decreased proteinuria after coenzyme Q10 supplementation therapy, while the other was not improved.

**Lessons:**

Based on the cases we reported and from the literature, recognition of *ADCK4* mutation through early and accurate genetic screening could be helpful in avoiding unnecessary toxicities and in preventing complications arising in mitochondrial nephropathy.

## 1 Introduction

Most mitochondrial cytopathies are involved in multiple organ systems and often present with prominent neurologic and myopathic deficits in childhood.^[1]^ Recent studies have found that mitochondrial dysfunction caused by genetic mutations in mitochondrial genes or nuclear genes can also lead to renal disease, for example, mitochondrial DNA A3243G mutation,^[2]^*PDSS2* gene,^[3]^*COQ2* gene,^[4]^ and *ADCK4* gene mutation.^[5]^ Mitochondrial nephropathy has a poor prognosis and often leads to the end-stage renal disease. At present, only rare cases reported about the mitochondrial nephropathy and the correlated genetic mutation.

Next-generation sequencing (NGS) provides coverage of more than 95% of the exons, which contain 85% of disease-causing mutations in mendelian disorders and many disease-predisposing single nucleotide polymorphisms (SNPs) throughout the genome.^[6]^ Here, we report 2 children with proteinuria in whom *ADCK4* mutation were identified (c.748G>C, p.D250H and c.532C>T, p.R178W in first patient, and c.625C>G, D209H and c.614C>T, p.S205N in second patient). The results of NGS analysis was further confirmed by Sanger sequencing. One patient showed a reduced proteinuria after coenzyme Q10 supplementation therapy, although the other patient showed no improvement of proteinuria and renal injury.

## 2 Case presentation

### 2.1 Ethical approval

This study was approved by the Ethical Committee, Children's Hospital, Zhejiang University School of Medicine. This case report was prepared in accordance with the Health Insurance Portability and Accountability Act (HIPAA) regulations. The patient's parents/legal guardians provided informed consent for data collection and publication.

### 2.2 Case 1

A 9-month-old girl was admitted to the hospital because of abnormal urine for 10 days, and fever for 2 days. No edema or rash was found in physical examination. Routine urine revealed proteinuria and microscopic hematuria; the result of 24-hours of proteinuria was 413.4 mg/24 h (45.9 mg/kg/24 h), the serum albumin was 40.3 g/L, the serum creatinine level was 30 μmol/L. Magnetic resonance imaging (MRI) showed the development of the brain was delayed. Renal MRI showed nothing abnormal.

She was born by normal delivery after an uncomplicated pregnancy. Her growth history showed mental developmental retardation. The patient had no history of liver, heart, or neuromuscular diseases. Her parents are both healthy without consanguineous marriage or similar disease history in their family.

### 2.3 Case 2

A 11-year-old girl was admitted to the hospital because of abnormal urine for more than 2 years. Because of the diagnosis of primary nephrotic syndrome at another hospital, she received the treatment of prednisone and tacrolimus late, but there was no relief in the symptoms for her. On physical examination, no sign of edema and rash was found. Routine urine and blood tests revealed proteinuria and micro-hematuria; the result of 24-hours of proteinuria was 2148.7 mg/24 h (55.5 mg/kg/24 h), the serum albumin was 38.5 g/L, the serum creatinine level was 54 μmol/L. Renal ultrasound showed that renal parenchyma echo enhancement. The pathology of renal biopsy revealed focal segmental glomerular sclerosis. Her medical history was unremarkable. Her parents are both healthy without consanguineous marriage and similar disease history in their family.

### 2.4 Next-generation sequencing

Genomic DNA was extracted from 5 mL peripheral blood of the patients and their parents, using a QIAamp Blood DNA mini Kit (Qiagen, Milano, Italy) according to the manufacturer's instructions. Concentrations of DNA were determined by NanoDrop spectrophotometer (ThermoScientific, Waltham, MA). DNA samples were stored at −20 °C until usage. Array capture was used to enrich the relevant human genes (SeqCap EZ Human Exome Library v2.0; Roche, Basel, Switzerland) and these genes were sequenced on the Illumina HiSeq 2000 plataform (2016 Illumina, Inc, San Diego, CA).

The following principal steps were taken to prioritize the high-quality variants: variants within intergenic, in tronic, and untranslated region (UTR) regions and synonymous mutations were excluded from downstream analysis; variants with quality score less than 20 were excluded; only the conservation score (phyloP) from comparison of human and 43 vertebrates higher than 3 were considered; and after this prior selection, the remaining genes were filtered by the function. The software PolyPhen-2 (http://genetics.bwh.harvard.edu/pph2/) predicted possible impact of variants. The final set of selected variants was visually inspected using an Integrative Genomics Viewer. Previously described polymorphic variants in public data were investigated and compared with the variations found in the current exome. The selected mutations to be investigated in each group of this study were not found in previous exome sequences (http://evs.gs.washington.edu/EVS/).

Sanger sequencing was used to confirm the NGS data. DNA from the patient and her parents were submitted to the PCR. Polyacrylamide gel electrophoresis was used to determine the size of amplification products. Products were purified using the QIAquick PCR purification kit (Qiagen) and then submitted to sequencing reaction using both forward and reverse primers with the ABI BigDye Terminator Cycle Sequencing Kit v. 3.1 on an ABIPRISM 3730XL Genetic Analyzer (Applied Biosystems, Foster City, CA). The results were aligned to the reference sequence, and mutations were identified with Sequencer software (http://www.genecodes.com). All primers were designed using the online tool Primer3 (Table 1).

**Table 1:**
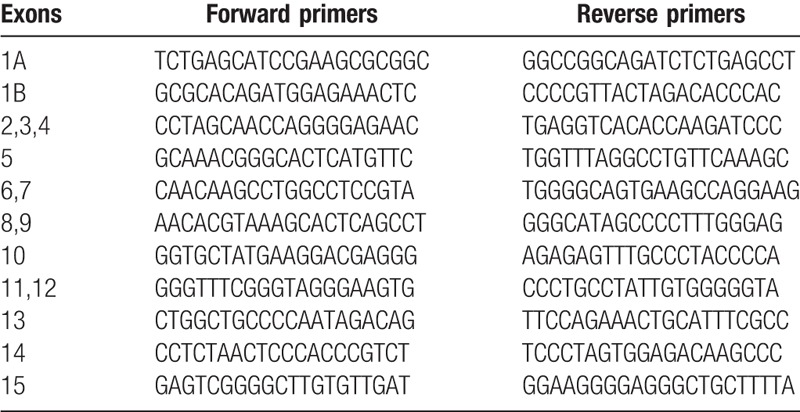
Primers needed for each exon of *ADCK4* gene.

Protein and DNA sequence alignments were performed using the ClustalW and the MultAlin (http://multalin.toulouse.inra.fr/multalin/), respectively. The prediction of amino acid substitution on the biological function of the protein was evaluated using both PolyPhen-2 and Provean software (http://provean.jcvi.org and http://genetics.bwh.harvard.edu/pph2/, respectively).

**Figure 1:**
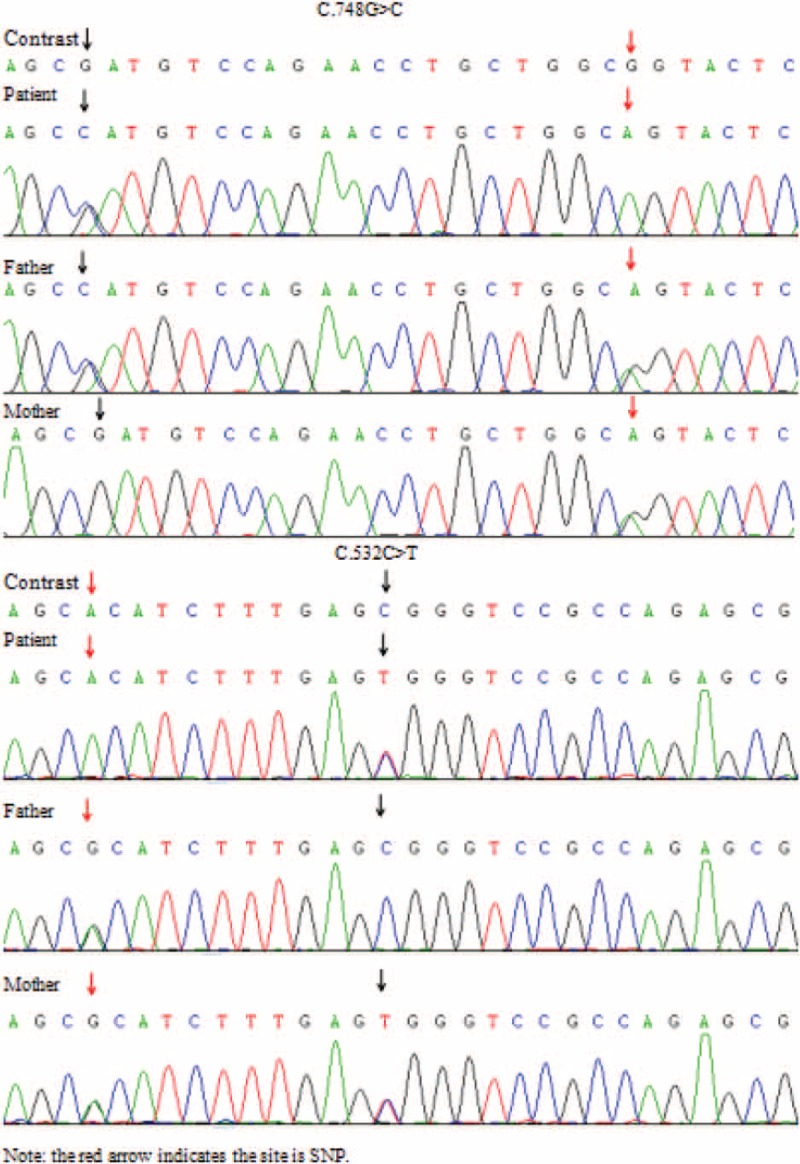
Case 1 sequencing analysis demonstrating the detection of c.748G>C (p.D250H) mutation in exon 9 and c.532C>T (p.R178W) mutation in exon 7 of the *ADCK4* gene.

**Figure 2:**
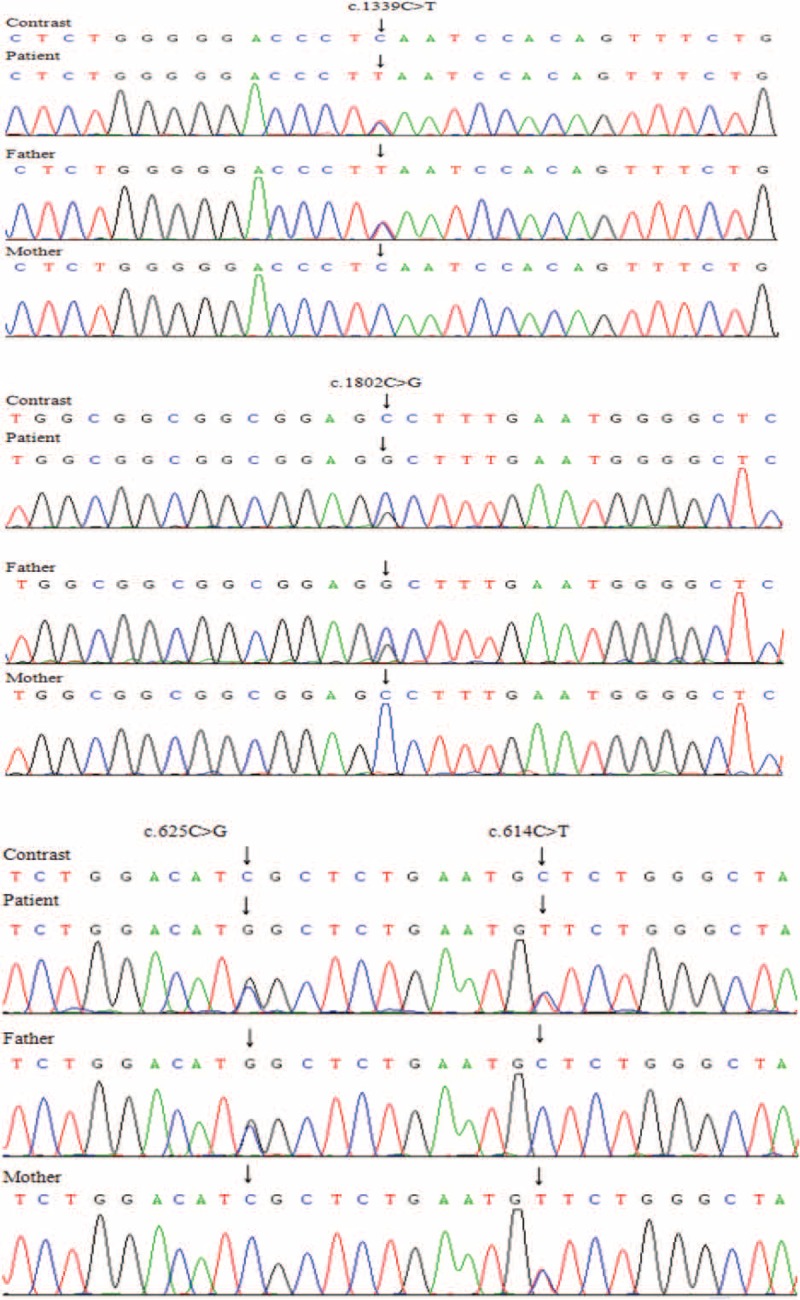
Case 2 sequencing analysis demonstrating the detection of c.1802C>G (p.G601A) mutation in exon 14 and c.1339C>T (p.E447K) mutation in exon 11 of the *NPHS1* gene, c.625C>G (D209H) mutation in exon 8 and c.614C>T (p.S205N) mutation in exon 8 of the *ADCK4* gene.

**Figure 3:**
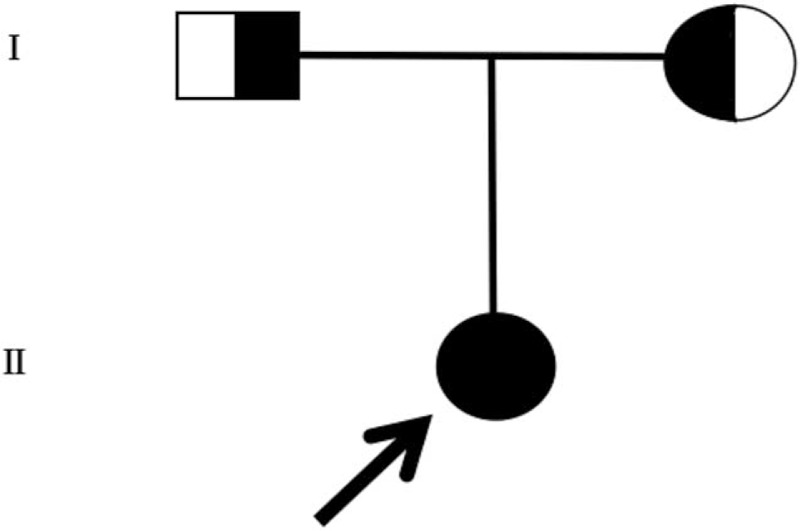
Pedigree of the family carrying the novel heterozygous mutation in *ADCK4* gene. The results of the analysis of the patients’ family members suggested an autosomal recessive model of inheritance.

The NGS analysis was performed on the patients and their parents, Case 1 revealed with heterozygous *ADCK4* mutation: c.748G>C (p.D250H) (MAF = 0) and c.532C>T (p.R178W) (rs398122978, MAF = 0) and confirmed by Sanger sequencing again. Her father carried the mutation of c.748G>C (p.D250H), and her mother carried the mutation of c.532C>T (p.R178W), respectively (Fig. 1). We also identified other genetic genes mutations about this patient, such as *ARHGEF6* gene, *ARID1A* gene, and *SETBP1* gene, which were related to mental retardation. But these mutations cannot contribute to proteinuria. Case 2 revealed heterozygous c.1802C>G (p.G601A) (rs114615449, MAF = 0.004) and c.1339C>T (p.E447K) (rs28939695, MAF = 0.007) mutations in *NPHS1* gene and heterozygous c.625C>G (D209H) (MAF = 0) and c.614C>T (p.S205N) (MAF = 0) mutation in *ADCK4* gene, which also confirmed by Sanger sequencing again. Family analysis showed that the patient had the same genotype in *NPHS1* gene mutation with her father (her father remains fine and no signs for nephropathy was found), and her *ADCK4* gene genotype were inherited from her father and mother, respectively. Her father carried the mutation of c.625C>G (p.D209H), and her mother carried the mutation of c.614C>T (p.S205N) (Fig. 2) in *ADCK4* gene. In addition to these 2 genes, we also identified other mutations in case 2, such as *TMEM237* gene, *C9* gene, *SCNN1G* gene, *KL* gene, and so on, which could not be linked to the disease phenotype. Both of the parents denied the history of proteinuria. The results of the analysis in the patients’ family members revealed an autosomal recessive model of inheritance in this disease (Fig. 3).

### 2.5 Treatment and follow-up

**Figure 4:**
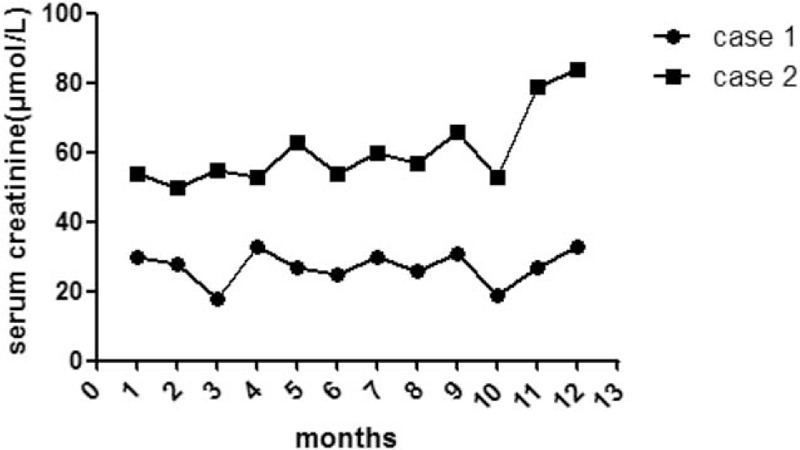
Variation of serum creatinine (μmol/L) within 1-year follow-up in 2 patient with proteinuria.

Case 1 and case 2 were both given the Coenzyme Q10 supplementation therapy in dose 15 to 30 mg/kg/d. After 12 months follow-up, the urine protein of case 1 turned to negative, and serum creatinine and urea nitrogen remains normal. However, case 2 still had gross proteinuria, serum creatinine, and urea nitrogen was increased gradually (Fig. 4).

## 3 Discussion

Mitochondrial disease is one of the most rare and complex genetic diseases, and its inheritance is related to maternal inheritance of mitochondrial genes and Mendel inheritance of nuclear genes.^[7]^ Studies have shown that mitochondrial dysfunction caused by either mitochondrial genes or nuclear genes mutations, may damage the kidney.^[8,9]^ Mitochondrial nephropathy can be characterized by glomerular lesion, renal tubular dysfunction, interstitial nephritis, and cystic renal disease.^[10,11]^ The mutation of Mitochondrial genes and nuclear genes encoding mitochondrial proteins can cause isolated glomerular lesions, manifesting proteinuria or nephrotic syndrome, though some patient accompanied by extra-renal symptoms.^[12]^ The renal pathology often are FSGS, and do not respond to steroid therapy and immunosuppressive therapy, some patients are benefit from the therapy of coenzyme Q10, which affect the synthesis of coenzyme Q10 gene mutation.^[5]^

According to literature, *ADCK4* localizes in mitochondria and foot processes in podocytes that interacts with *COQ6* endogenously.^[5]^ The *ADCK4* gene (OMIM 615567) locates in chromosome 19p13.2 and contains 15 exons spanning approximately 12 kb of DNA. *ADCK4* disease typically manifests as an isolated nephropathy with occasional extrarenal symptomatology on contrast to the mutations in *PDSS2*, *COQ2*, and *COQ6*, those renal symptoms usually occur as part of a multisystemic disease complex encompassing progressive encephalopathy, seizures, and hypertrophic cardiomyopathy.^[13]^ In patients with *ADCK4* mutant, advanced chronic kidney disease (CKD) at time of diagnosis was more prevalent than in patients with NPHS2 mutation. Of patients with *ADCK4* disease, 38.5% presented with CKD stage 5, compared with 15.6% of patients with WT1 mutation and 2.9% of patients with *NPHS2* mutation at time of diagnosis.^[14]^

CoQ10 is an essential component of eukaryotic cells and is involved in crucial biochemical reactions such as the production of ATP in the mitochondrial respiratory chain, the biosynthesis of pyrimidines, and the modulation of apoptosis.^[15]^ The biosynthesis of CoQ10 requires at least 13 different genes. Mutations in these genes may cause primary CoQ10 deficiency, a clinically and genetically heterogeneous disorder. Primary CoQ10 deficiency was first described in 1989 but only in the last decade the molecular bases of this disorder have been elucidated.^[16]^ To date, mutations in 8 genes (*PDSS1*, *PDSS2*, *COQ2*, *COQ4*, *COQ6*, *ADCK3*, *ADCK4*, and *COQ9*) have been associated with CoQ10 deficiency presenting with a wide variety of clinical manifestations.^[17,18]^ It is of great importance that physicians should promptly recognize these disorders because most patients respond to oral administration of CoQ10.

We herein presented case 1 with onset at early infancy, and manifested by proteinuria and mental retardation. Genetic testing revealed mutation results fit with compound heterozygosity model: c.748G>C (p.D250H) and c.532C>T (p.R178W) mutation in the *ADCK4* gene. The second patient's renal pathology was FSGS, and showed no response to steroid therapy and immunosuppressive therapy. Genetic testing also revealed mutation results fit with compound heterozygosity model: c.625C>G (D209H) and c.614C>T (p.S205N) mutations in *ADCK4* gene. From these mutations, c.614C>T (p.S205N) is a novel missense which was never reported before (Table 2).^[5,14,20,21]^ Further analysis finds that this mutation is harmful to protein structure related to this disease.

**Table 2:**
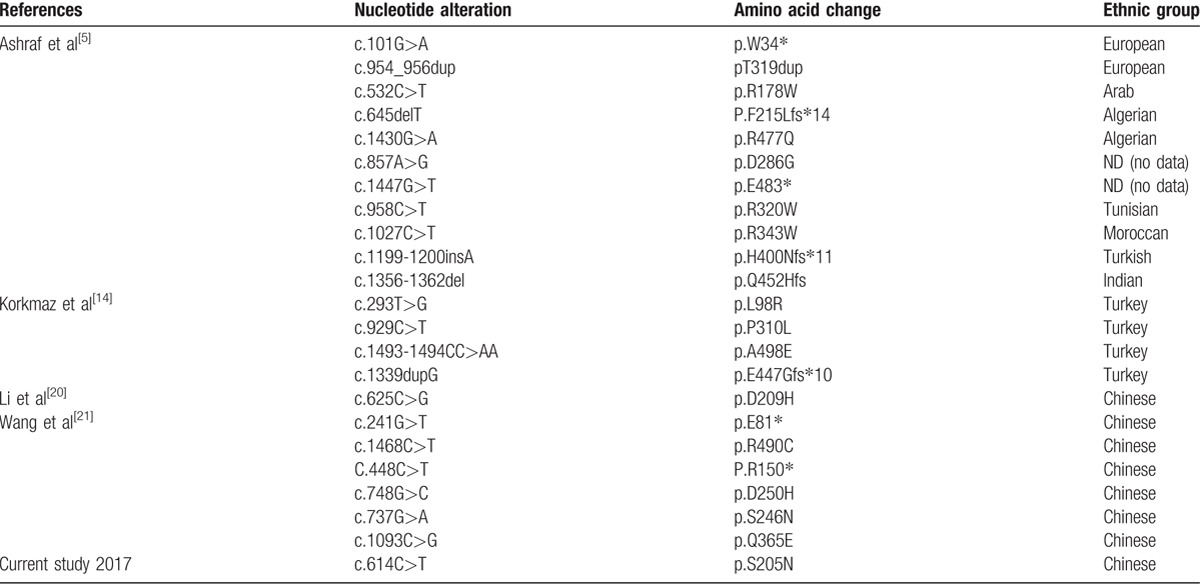
Identified mutations in patients with *ADCK4*.

According to the clinical manifestation and genetic analysis, those 2 cases were diagnosed as mitochondrial nephropathy. Mitochondrial nephropathy caused by *ADCK4* mutation is unresponsive to steroid and immunosuppressive therapy, and can rapidly progress to end-stage renal failure, and finally requires renal replacement. Clinical symptoms can be relieved in some patients with coenzyme Q10 administration. In this report, case 1 was not given steroid therapy, and only enough dose of coenzyme Q10 treatment was able to control the proteinuria and she revealed full response to CoQ10 therapy. Case 2 showed no response to steroid, immunosuppressive agents and CoQ10 administration together, and her glomerular filtration rate deteriorated progressively. Both patients we diagnosed with CKD, after 12 months follow-up, the glomerular filtration rate of case 1 is normal, while case 2 presented with CKD stage 2.

Ashraf et al^[5]^ reported that 1 of 15 patients with SRNS and a homozygous *ADCK4* frameshift mutation had partial remission following CoQ10 treatment. Korkmaz et al^[14]^ reported 2 of 26 cases with early stage *ADCK4* nephropathy demonstrated a decrease of proteinuria by 50% and 80%, respectively, within 6 weeks of treatment of CoQ10 supplementation soon after diagnosis. To date, only these 2 studies have reported the response to CoQ10 treatment of patients with *ADCK4* mutations. Furthermore, 1 patient reported by Park et al^[19]^ showed complete remission of proteinuria with cyclosporine treatment, had recently started CoQ10 supplementation (30 mg/kg/d) with simultaneously tapering doses of oral steroid and cyclosporine. One of our patients received CoQ10 supplementation when she was 9 months only and the proteinuria disappeared gradually. In the meantime, another patient received CoQ10 supplementation when she was 11 years old and demonstrated no response. Combined with these and our data, it is implied that early recognition for *ADCK4* mutation and early CoQ10 supplementation to patient would be necessary for patients with full or partial response to CoQ10 therapy and benign prognosis in future.

## 4 Conclusions

In conclusion, we reported 2 cases of mitochondrial nephropathy with *ADCK4* gene mutation, and one respond to the CoQ10 therapy but the other did not. Although this is a rare disease, it is one of the important causes of the end-stage renal failure in childhood. Early detection and early intervention of this disease could contribute to the prevention of progress of CKD in children.
